# Cobalamin and Folate Status in 6 to 35 Months Old Children Presenting with Acute Diarrhea in Bhaktapur, Nepal

**DOI:** 10.1371/journal.pone.0090079

**Published:** 2014-03-03

**Authors:** Manjeswori Ulak, Ram K. Chandyo, Ramesh K. Adhikari, Pushpa R. Sharma, Halvor Sommerfelt, Helga Refsum, Tor A. Strand

**Affiliations:** 1 Centre for International Health, University of Bergen, Norway; 2 Department of Child Health, Institute of Medicine, Kathmandu, Nepal; 3 Department of Pediatrics, Kathmandu Medical College, Nepal; 4 Community Medicine, Kathmandu Medical College, Nepal; 5 Department of Nutrition, Institute of Basic Medical Sciences, University of Oslo, Norway; 6 Medical Microbiology, Innlandet Hospital Trust, Lillehammer, Norway; University of Melbourne, Australia

## Abstract

**Background:**

Cobalamin and folate are essential micronutrients and are important in DNA and RNA synthesis, cell proliferation, growth, hematopoiesis, and cognitive function. However, data on cobalamin and folate status are lacking particularly from young children residing in low and middle income countries.

**Objective:**

To measure cobalamin and folate status and identifies their predictors among 6 to 35 months old children presenting with acute diarrhea.

**Design:**

This was a cross-sectional study in 823 children presenting with acute diarrhea. We measured plasma cobalamin, folate, methylmalonic acid and total homocysteine who sought treatment for acute diarrhea between June 1998 and August 2000.

**Results:**

The mean (SD) plasma concentrations of cobalamin, folate, total homocysteine and methylmalonic acid were 206 (124) pmol/L, 55 (32) nmol/L, 11.4 (5.6) µmol/L and 0.79 (1.2) µmol/L, respectively. The prevalence of low plasma cobalamin (<150 pmol/L) was 41% but less than 2% (15) children had low folate concentration (<10 nmol/L). Plasma homocysteine and methylmalonic acid concentrations were negatively associated with cobalamin concentration but not associated with folate status. The prevalence of cobalamin deficiency was higher in breastfed than non-breastfed children (44% vs 24%; *p* = <0.001). The prevalence of hyperhomocysteinemia (>10 µmol/L) and elevated methylmalonic acid (>0.28 µmol/L) were 73% and 52%, respectively. In the regression analyses, the plasma cobalamin concentration was positively associated with age, and introduction of animal or formula milk.

**Conclusions:**

Our study indicated that poor cobalamin status was common particularly among breastfed children. Folate deficiency was virtually none existent. Possible consequences of cobalamin deficiency in young children need to be explored.

## Introduction

Cobalamin and folate are essential nutrients for young children and is important for DNA and RNA synthesis, cell proliferation growth, hematopoiesis and cognitive function. Recent studies suggest that deficiencies of these nutrients are common in young children [1], [2]. However, the status of cobalamin and folate in many low and middle-income countries (LMIC) including Nepal is still not known.

The main source of vitamin B12 is animal-derived foods, which are expensive and for cultural and religious reasons often not eaten at all [3]. The recommended daily allowances (RDA) of cobalamin for young children range form 0.5–0.9 µg/day [4] which is not possible to obtain from a strictly vegetarian or lacto-vegetarian diet [5], [6]. On the other hand, folate is abundant in vegetarian based diets such as in lentils, beans and green vegetables [7].

Deficiencies of both folate and cobalamin can result in an increase in the total homocysteine (tHcy) concentration [8] which is associated with an increased risk for abortion, birth defects, low birth weight [9], [10] and cardiovascular diseases [11]. Cobalamin deficiency also leads to an elevation in methylmalonic acid (MMA) concentration due to its role in the conversion of methylmalonyl CoA to succinyl CoA [7], [8]. Early stages of cobalamin and folate deficiencies can be detected by analyzing MMA and tHcy concentrations. Megaloblastic anemia occurs in both of these vitamin deficiencies but usually only when it is more pronounced. Cobalamin deficiency may also cause neuro-psychiatric disorders and result in cognitive impairment [12], [13].

In an antenatal clinic based study from Kathmandu valley, 61% had elevated serum MMA, and 49% had low serum cobalamin concentration [14]. Similar findings were also observed among non-pregnant women of reproductive age in Bhaktapur [15] and in a study in southern Nepal [16]. The cobalamin status was found poor in infants whose mothers consumed a predominantly vegetarian diet, as maternal cobalamin status is reflected in the breast milk [17]–[19]. The common staple diet in Nepal particularly for young children is mostly vegetarian and seldom includes meat or meat products [20]. There are few population-based studies in LMIC that include tHcy and MMA to assess cobalamin and folate status. In this paper, we report cobalamin and folate status and its predictors in children 6–35 months of age with acute diarrhea residing in the peri-urban Bhaktapur community in Nepal. All children residing in the defined area, who had diarrhea, and in the right age range were eligible for participation.

## Subjects and Methods

### Study site and population

The study was conducted in Bhaktapur municipality and its surrounding suburbs, which is located 15 km east of the capital Kathmandu. Bhaktapur is situated about 1400 meter above sea level and there is a cold and dry winter from November to March, the summer months are generally hot and humid, and the autumn is relatively warm. The municipality has approximately 84,000 inhabitants; Bhaktapur is the most densely populated district in Nepal [21]. The local people mostly practice Hinduism or Buddhism and about 88% belong to the Newar ethnic group and are mainly engaged in work related to agriculture. In the study area, there are some migrants mainly belonging to the Tamang and Lama ethnic groups, most of them work in the carpet factories. Rice is the main staple food in this community and grown on their own land whereas consumption of lentils and green or dry vegetables varies with season. Although most of the people in the study are non-vegetarian, consumption of meat products is rare and depends on availability, which may be occasionally higher during festivals and ceremonies. The recruitment of study subjects was done in Siddhi Memorial Children's Hospital, which is a non-profit private organization providing 24-hour pediatric services in this community.

### Study design

**Figure 1:**
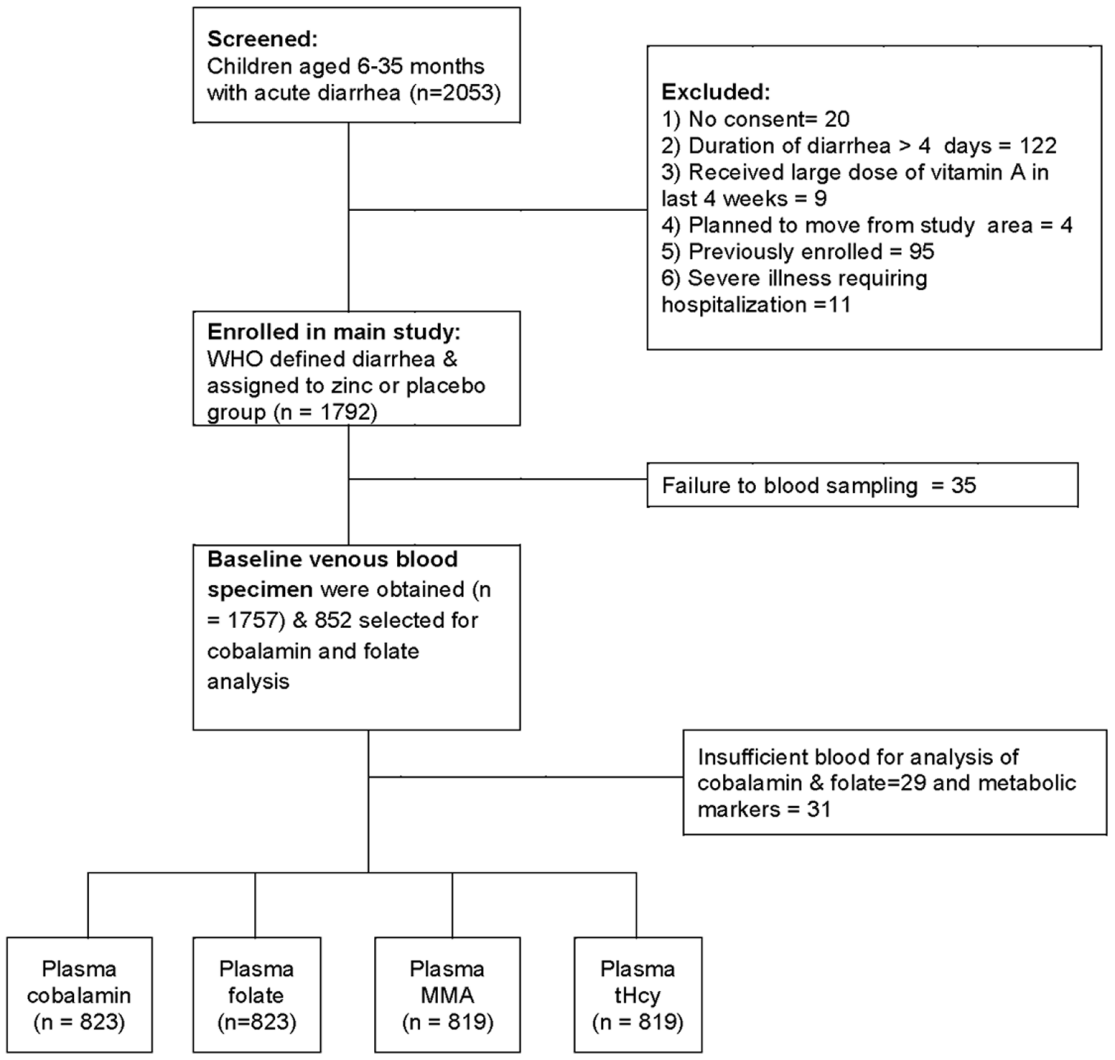
Flow chart of recruitment of children, blood sampling and analysis.

This was a cross-sectional study and included children 6 to 35 months with acute diarrhea, participating in a double blind randomized clinical trial, aimed to measure the effect of zinc administration during an episode of diarrhea [22]. The study was conducted from June 1998 to September 2000. Children were identified through weekly surveillance or spontaneous visits to our field clinic and were screened by a physician. Diarrhea was defined according to the WHO guidelines as a passage of three or more loose or watery stools in past 24 hours with a recent change in stool consistency. A total of 1,792 eligible children were enrolled in the main study and informed written consent was obtained from one of the parents. Baseline venous blood was collected from 1,758 children before initiation of the intervention. We randomly selected approximately half of the enrolled children (852) for analysis of folate and cobalamin status. The current analysis is based on 823 children from whom we had obtained sufficient amounts of blood to analyze plasma cobalamin, folate, MMA and tHcy concentrations. Exclusion criteria and selection of subjects for cobalamin and folate status are shown in **Figure 1**.

### Data collection and blood sampling

All baseline and morbidity information was recorded on the day of enrollment. The children were weighed using a scale to the nearest 100 g (Salter, SECA, Germany) and length or height was measured using a locally made length board to the nearest ± 0.1 cm. A study physician collected blood from each child's cubital vein into heparinized polypropylene tubes (Monovette; Sarstedt, Numbrecht, Germany), mainly between 9 AM and 1 PM. The hemoglobin concentration was measured with a HemoCue® Hb 201^+^ hemoglobinometer (HemoCue AB, Ängelholm, Sweden) immediately after blood sampling according to the manufacture's instruction. The blood was then centrifuged at 760×g for 10 min in room temperature, and plasma was divided into polypropylene vials (Eppendorf, Hinz, Germany) and wrapped immediately by aluminum foil to prevent from light and stored at −40°C until transfer to Norway on dry ice for analysis. Then the specimens were stored at −70°C for 3–4 years and analyzed at the pharmacology laboratory, Oxford University, UK. After thawing, cobalamin and folate (n = 823) were analyzed by microbiological assays using a chloramphenicol-resistant strain of Lactobacillus casei and colistin sulfate-resistant strain of *Lactobacillus leichmannii*, respectively [23], [24]. These assays were adapted to a microtiter plate format and carried out by a robotic workstation [25]. Plasma concentrations of MMA and tHcy (n = 819) were analyzed by a modified gas chromatography-mass spectrometry method based on ethylchloroformate derivatization [26].Total homocysteine was analyzed by using commercial kits from Abbott Laboratories [27].

### Definitions

We used the WHO definitions of cobalamin and folate deficiency which is <150 pmol/L of plasma cobalamin and <10 nmol/L of plasma folate [28]. We defined marginal cobalamin deficiency as being present when the concentration was between 150 to 200 pmol/L. There are no reference limits available tHcy and MMA in children. So we defined hyperhomocysteinemia when plasma tHcy concentration was >10 µmol/L and elevated methylmalonic acid when the plasma MMA concentration was >0.28 µmol/L. Hemoglobin concentration was adjusted on altitude, as partial pressure of oxygen is decreased in the higher altitude. Thus, we added 0.3 g/dL to the standard cut off values of Hb for the 1400 meter altitude of the study area thus anemia was defined as having Hb concentration <11.3 g/dL [29].

### Data entry and statistical analysis

All forms were checked manually by supervisors and physicians for completeness and consistency. The data was then double entered into Microsoft Visual FoxPro databases with computerized logic, range and consistency checks. Weight for age, length/height for age, and weight for length/height Z-scores were calculated using the most recent WHO growth charts [30]. Statistical analyses were undertaken using Stata®, version 10 (Stata Corp, College Station, TX). We presented mean, median or interquartile ranges (IQR) of cobalamin, folate, tHcy and MMA according to age group and breastfeeding status. We used chi square to test differences in prevalence between groups. Spearman rank correlation analyses was used to measure crude associations between plasma concentrations of cobalamin, folate, MMA and tHcy. A *P* value of <0.05 was considered to reflect statistical significance. Simple and multiple regression analyses were used to examine the associations between the dependent variables (cobalamin, folate, MMA and tHcy) and different independent variables. The variables listed in **Table 1** including breastfeeding status (yes or no), age, maternal and paternal age and education, weight for age, length/height for age and weight for length/height Z-scores, family size, ownership of land, loose or watery stool, stool frequency, dehydration and intake of milk and other foods etc. were included in the initial assessments. We used a stepwise process to select the variables for the final model using cut off of *P* value <0.15 as described elsewhere [31]. The interaction between various potential predictors on markers of cobalamin and folate status was also analyzed by inclusion of interaction terms in the multiple regression models. We also assessed the linearity between the continuous independent (listed in Table 1) and dependent variables in the regression models using generalized additive models in R *(* http://www.r-project.org *)* adjusting for the same variables as in the multiple linear regression models.

**Table 1:** Variables assessed in the multivariable linear regression models measuring predictors for plasma folate, cobalamin, homocysteine, or methylmalonic acid in 823 Nepalese children 6 to 35 months of age.

Variables	Continuous	Categorical
Breastfeeding		yes/no
Age	months	Infants: yes/no
Age x breastfeeding interaction		Breastfeeding: yes/no
Gender		male/females
Living in joint families		yes/no
Members in the household	number	
Years of schooling, mother	Years	school: yes/no
Years of schooling, father	Years	school: yes/no
Weight for age Z score (WAZ)	Z scores	<-2 WAZ: yes/no
Weight for length Z score (WHZ)	Z scores	<-2 WHZ: yes/no
Height for age Z score (HAZ)	Z scores	<-2 HAZ: yes/no
Maternal age	Years	
Paternal age	Years	
Ownership of land		yes/no
Stool frequency	Number, past 24 hours	
Animal or formula milk use regularly		yes/no
Introduction of semi-solid or solid foods		yes/no
Season		three categories
Dehydration		yes/no

### Ethical considerations

The study had ethical clearances from the Nepal Health Research Council, Kathmandu. The implementation of all aspects of the project was in agreement with the International Ethical Guidelines for Research Involving Human Subjects as stated in the latest version of the Helsinki Declaration. Informed written consent was obtained from at least one of each child's parents.

## Results

The baseline characteristics of the study population are presented in **Table 2**. A total of 823 children were included for cobalamin and folate biomarker analyses; the mean age was 15.7 months and 465 (56%) were boys. Most of the children [673 (82%)] were breastfed at the time of enrollment and more than half were living in joint families [457 (55.5%)]. The prevalence of stunting, underweight, and wasting (<-2 Z score of length or height for age, weight for age and weight for length or height) were 34%, 25% and 10% respectively. About half (52%) of the 674 children in whom we measured hemoglobin concentration had anemia. The mean duration of diarrhea before enrollment was 3.1 days, 11% children had some signs of dehydration and 12% children had dysentery. Sixty-nine children (8.3%) had fever (axillary temperature >100°F) and 322 (39%) children had cough and/or difficult breathing.

**Table 2:** General and clinical characteristics of 823 study children with acute diarrhea in Bhaktapur, Nepal.

Characteristics	Values^1^
Mean age of child in months, (SD)	15.7±7.6
Boys	465 (56)
Breastfed	673 (82)
Animals or formula milk fed	279 (34)
Semisolid or solid food	791 (96)
Living in joint family	457 (56)
Ownership of land	596 (72)
< -2 Z-score weight for age	210 (25)
< -2 Z-score length/height for age	282 (34)
< -2 Z-score weight for length/height	81 (10)
Mean age of mother in year	25 (4.6)
Mean schooling of mother in year, (SD)	2.6±3.4
Mean schooling of father in year, (SD)	6.7±3.6
History of fever in last 24 hour prior to enrollment	236 (29)
Body temperature >100°F	69 (8)
History of cough and or difficulty breathing in last 24 hour prior to enrollment	322 (39)
Mean duration of diarrhea before enrollment, days (SD)	3.1±1.1
Children with blood in stool in last 24 hour prior to enrollment	99 (12)
Children with dehydration according to WHO/IMCI	93 (11)
Mean plasma cobalamin (pmol/L) (SD)	206±125
Mean plasma folate (nmol/L) (SD)	55±32
Mean plasma total homocysteine (µmol/L) (SD)	11.4±5.6
Mean plasma methylmalonic acid (µmol/L) (SD)	0.79±1.2
Mean hemoglobin (g/dL)^2^ (SD)	11.1±1.1
Anemia^3^ (hemoglobin <11.3 g/dL)	352 (52)

1 *Values are number (%) unless otherwise mentioned. ^2^Hemoglobin was measured from 674 subjects.*

3 *Altitute adjusted hemoglobin cut off value.*

### Plasma cobalamin, folate and their metabolic markers according to age group and breastfeeding status

The mean (SD) cobalamin, folate, total homocysteine and methylmalonic acid concentrations were 207 (125) pmol/L, 55 (32) nmol/L, 11.4 (5.6) µmol/L and 0.79 (1.2), µmol/L respectively. The median (interquartile range; IQR) cobalamin concentration was lower in breastfed (147; 103-239 pmol/L) than in non-breastfed children (379; 314–399 pmol/L) among 6–11 months old infants (*p* = 0.003). In the same age group, the median (IQR) folate was higher in breastfed (65.6; 46.6–93.0 nmol/L) than in non-breastfed (35.5; 22.8–48.8 nmol/L; *p* = 0.005) infants and it declined with increasing age. The median plasma MMA and tHcy concentrations were also higher among those who were breastfed than in those who were not (MMA of 0.52 vs 0.42 µmol/L, *p* = 0.007 and tHcy of 12.4 vs 7.2 µmol/L, *p* = <0.001) (**Table 3**).

**Table 3:** Plasma cobalamin, folate, methylmalonic acid and total homocysteine concentrations in different age groups according to breastfeeding status of the study children in Bhaktapur, Nepal.

	Breastfed			Not breastfed		
	Age (months)			Age (months)		
	06 to 11	12 to 23	24 to 35	06 to 11	12 to 23	24 to 35
	**Cobalamin**					
Median (pmol/L)	147	181	195	379	231	231
IQR (pmol/L)	103–239	119–264	128–253	314–399	159–349	148–306
Number	315	318	40	6	24	120
	**Folate**					
Median (nmol/L)	65.6	49	29.7	35.5	30.78	20.6
IQR (nmol/L)	46.6–93.0	35.2–70.2	16.5–47.0	11.8–48.8	17.8–43.2	13.6–32.7
Number	315	318	40	6	24	120
	**Methylmalonic acid**					
Median (µmol/L)	0.52	0.42	0.42	0.42	0.49	0.38
IQR (µmol/L)	0.3–1.0	0.2–0.8	0.2–0.7	0.3–0.6	0.3–0.6	0.3–0.6
Number	310	319	40	6	24	120
	**Total homocysteine**					
Median (µmol/L	12.4	10	9.6	7.2	7.4	7.3
IQR (µmol/L)	9.2 –15.7	7.5–13	7.4–13	5.3–8.2	5.8–9.2	6.0–9.4
Number	310	319	40	6	24	120

### Proportion who were cobalamin or folate deficient (Table 4)

Low cobalamin concentration (<150 pmol/L) was found in 40.7% (335) and 56% had plasma cobalamin <200 pmol/L. Forty four percent (299) of the breastfed and 24% (36) of the non-breastfed children had a cobalamin concentration <150 pmol/L. The mean tHcy and MMA concentrations were also higher in those with low cobalamin than in those with adequate plasma cobalamin concentration (13.2 vs 9.1 µmol/L, *p* = <0.001 and 1.0 vs 0.5 µmol/L, *p* = <0.001 respectively). None of the children had a plasma folate concentration <5 nmol/L and only 15 children (2%) had a folate concentration of <10 nmol/L. Most of the children (n  = 14) with low folate were more than one years old. Only one (0.3%) child had both a cobalamin concentration < 150 pmol/L and a folate concentration <10 nmol/L.

**Table 4:** Cobalamin and folate concentrations in the study children according to age, gender, breastfeeding status and their metabolic markers.^1^

Characteristics	Plasma cobalamin (pmol/L)				Plasma folate (nmol/L)		
	<150	150–200	>200	*P* value^2^	<10	>10	*P* value^2^
**Total children**	335 (40.7)	129 (15.7)	359 (43.6)		15 (1.8)	808 (98.2)	
**Age**							
6–11 months	163 (48.6)	43 (33.3)	115 (32.0)	<0.001	1 (6.7)	320 (39.6)	<0.001
12–35 months	172 (51.4)	86 (66.7)	244 (68.0)		14 (93.3)	488 (60.4)	
**Gender**							
Male	193 (57.6)	80 (62.0)	192 (53.5)	0.2	10 (66.7)	455 (56.3)	0.4
Female	142 (42.4)	49 (38.0)	167 (46.5)		5 (33.3)	353 (43.7)	
**Breast feeding status**							
Breastfed	299 (89.3)	107 (83.0)	267 (74.4)	<0.001	4 (26.7)	669 (82.8)	<0.001
Non-breastfed	36 (10.7)	22 (17.0)	92 (25.6)		11 (73.3)	139 (17.2)	
**Plasma folate**							
<10 nmol/L	1 (0.3)	2(1.5)	12(3.3)	0.01	_	_	
≥10 nmol/L	334 (99.7)	127(98.5)	347(96.7)		_	_	
**Plasma cobalamin**							
<150 pmol/L	_	_	_		1 (6.7)	334 (41.3)	0.007
≥150 pmol/L	_	_	_		14 (93.3)	474 (58.7)	
**Plasma tHcy** 3	14.0±6.4	11.9±5.7	9.1±3.7	<0.001	9.7±4.5	11.4±5.7	
>10 µmol/L	240 (72.3)	62 (48.4)	127(35.5)	<0.001	8 (53.3)	384 (47.8)	0.6
<10 µmol/L	92(27.7)	66 (51.6)	231(64.5)		7(46.7)	419 (52.2)	
**Plasma MMA** 3	1.0±1.5	0.7±1.2	0.5±0.6	<0.001	0.7±0.4	0.8±1.2	
>0.28 µmol/L	280 (84.7)	95 (74.2)	223 (62.3)	<0.001	13 (86.7)	585 (72.8)	0.2
<0.28 µmol/L	52 (15.3)	33 (25.8)	135 (37.7)		2 (13.3)	218 (27.2)	

1 *Values are number (%) unless otherwise mentioned*

2 *P values were obtained from Chi-square test or t-test*

3 *Plasma tHcy and MMA were analysed from 819 subject.*

**Figure 2:**
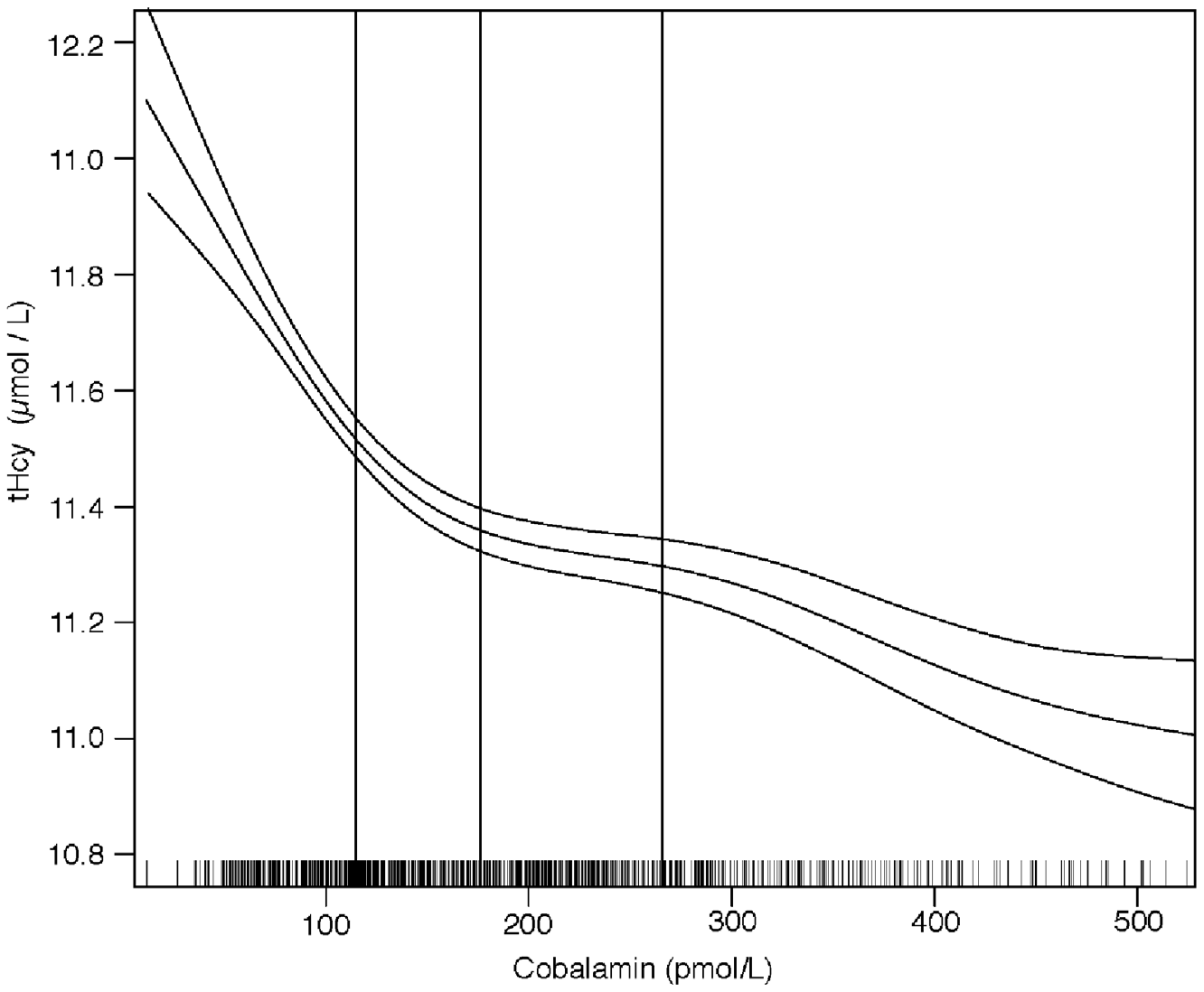
Association between plasma cobalamin and total homocysteine (tHcy) and methylmalonic acid (MMA) concentrations. The graphs were made by using generalized additive models in R. The upper and lower horizontal lines represent 95% CIs of regression line. The small bars on the x-axis show the distribution of individual observation and the vertical lines indicate the 25^th^, 50^th^ and 75^th^ percentiles of the plasma cobalamin.

The associations between plasma tHcy or MMA and cobalamin are depicted in **Figure 2** **and** **3** **.** The cobalamin concentration was negatively associated with MMA (r  = −0.35, *p* = <0.001) and tHcy (r = −0.47, *p* = <0.001]. The graphs indicate that MMA and tHcy levels start to rise when the cobalamin concentration drops below 150–200 pmol/L.

**Figure 3:**
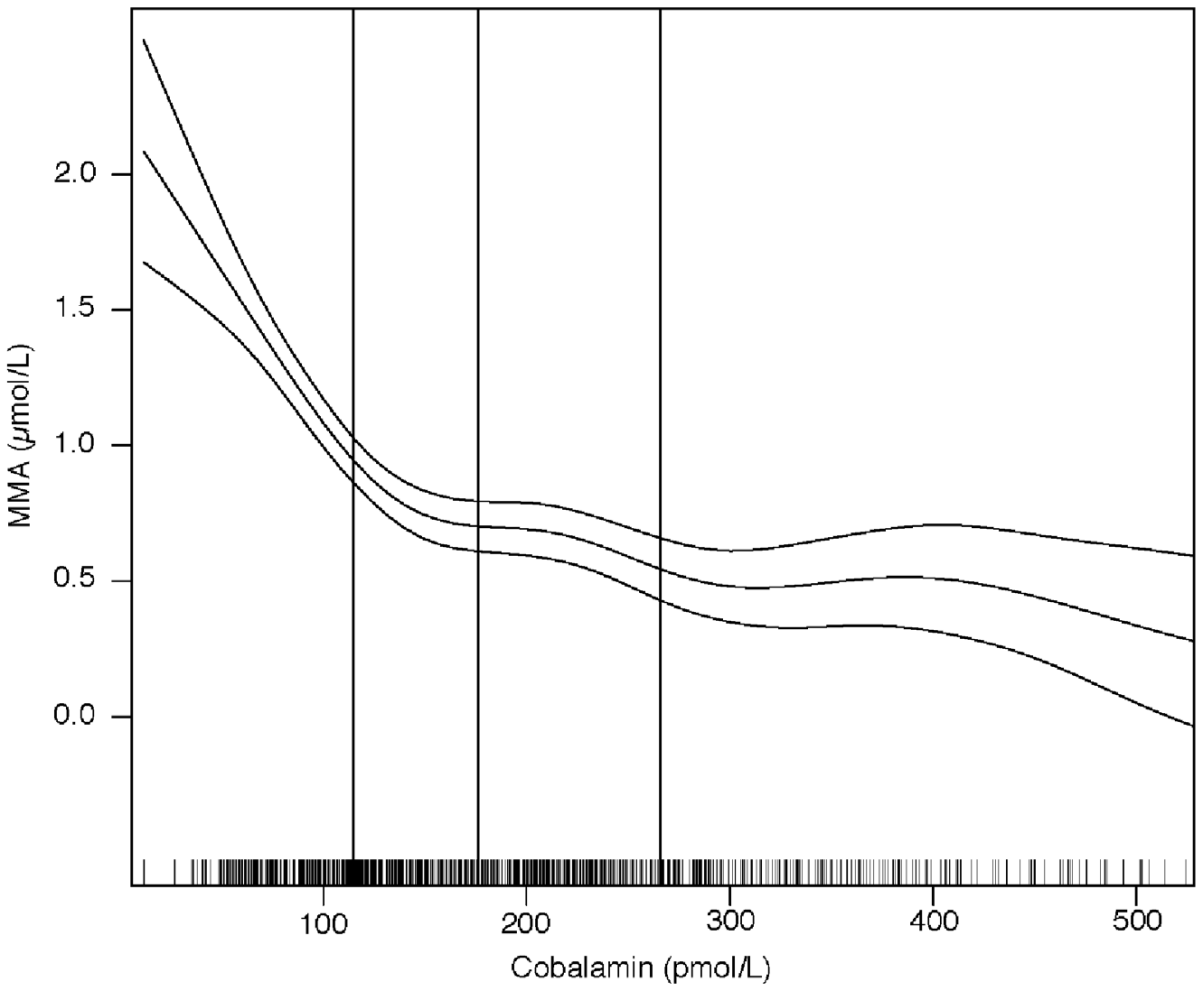
Association between plasma cobalamin and methylmalonic acid (MMA) concentrations. The graphs were made by using generalized additive models in R. The upper and lower horizontal lines represent 95% CIs of regression line. The small bars on the x-axis show the distribution of individual observation and the vertical lines indicate the 25th, 50th and 75th percentiles of the plasma cobalamin.

### Predictors for cobalamin and folate status

The results from the multiple linear regression analyses (**Table 5**) show that the plasma cobalamin concentration was significantly higher in non-breastfed children [adjusted mean difference 29.7 (95% CI: 0.27, 59.2) pmol/L] and positively associated with age [adjusted mean difference 2.0 (95% CI: 0.3, 3.4) pmol/L]. It was higher in children whose parents did not own land and in children who were dehydrated at enrollment. The concentration of plasma cobalamin was lower in those who did not consume animal or formula milk compared to those who did [adjusted mean difference −43.0 (95% CI: −60.8, −25.1) pmol/L]. The plasma folate concentration was negatively associated with age but in breastfed children only (*p* for interaction 0.002). The folate concentration was higher in those who did not consume animal or formula milk regularly [adjusted mean difference 8.6 (95% CI: 4.6, 12.5) nmol/L] and it was lower by −10.2 (95% CI: −16.1, −4.4) nmol/L in non-dehydrated children compared to those who were dehydrated. We also found a significantly higher plasma folate concentration during the spring season [adjusted mean difference 7.3 (95% CI: 3.3, 11.5) nmol/L] than in the summer. MMA concentration was negatively associated with age. Plasma tHcy was also negatively associated with age, however, only in breastfed children (*p* interaction <0.001) and it was lower in the spring than during the summer. Both cobalamin and folate and their metabolic markers were not significantly associated with anthropometric measurements or with clinical parameters listed in **Table 1**.

**Table 5:** Predictors for plasma cobalamin, folate, methylmalonic acid & total homocysteine concentrations among Nepalese children with acute diarrhea in Bhaktapur, Nepal.

Variables	Plasma cobalamin (pmol/L)			Plasma folate (nmol/L)			Methylmalonic acid (µmol/L)			Homocysteine (µmol/L)		
	Coeff*	95% CI	*P-*value	Coeff*	95% CI	*P-*value	Coeff*	95% CI	*P-*value	Coeff*	95% CI	*P-*value
Breastfeeding status												
Yes												
No	29.7	(0.27, 59.2)	0.04		Interaction						Interaction	
Age	2.0	(0.3, 3.4)	0.01		Interaction		−0.01	(−0.03, −0.001)	0.04		Interaction	
Age in breast fed				−1.96	(−2.3, −1.6)	<0.001				−0.24	(−0.31, −0.2)	<0.001
Age in non-breastfed				−0.61	(−1.4, 0.15)	0.12				0.04	(−0.11, 0.19)	0.6
Age and breastfeeding interaction				1.3	(0.5, 2.1)	0.002				0.27	(0.11, 0.43)	<0.001
Ownership of land												
Yes												
No	27.5	(9.0, 46.0)	0.003				0.21	(0.02, 0.4)	0.02	1.34	(0.5, 2.1)	0.001
Dehydration												
Yes												
No	−28.0	(−54.1, −1.9)	0.03	−10.2	(−16.1, −4.4)	0.001						
Use of animal or formula milk												
Yes												
No	−43.0	(−60.8, −25.2)	<0.001	8.6	(4.6, 12.5)	<0.001						
Seasons of enrollment												
Jun-Sept												
Oct-Jan				0.07	(−5.0, 4.8)	0.9	0	(−0.24, 0.18)	0.8	−1.92	(−2.8, −0.95)	<0.001
Feb-May				7.3	(3.1, 11.5)	0.001	−0.18	(−0.37, −0.004)	0.04	−1.19	(−2.0, −0.36)	0.005

* Adjusted regression coefficient and P values obtained from linear regression model adjusted for the variables included in this table, R square values of plasma cobalamin, folate, tHcy and MMA are 0.083, 0.30, 0.15 and 0.02, respectively.

## Discussion

We assessed cobalamin and folate status and identified their predictors in 6–35 months old Nepalese children with acute diarrhea. We found that poor cobalamin status was affecting almost one in every second child, which was more common in breastfed than in non-breast infants. Similar findings have been reported from studies conducted in India [2] and in other countries [32]–[34]. Infants who consumed animal or formula milk had higher plasma cobalamin concentrations than those who didn't, as was observed among Norwegian healthy children who were given animal milk [32]. This is most likely because the content of cobalamin in animal milk is 10 times higher than in breast milk [35]. Animal milk, particularly buffalo or cow's milk, or in the form of milk-tea (diluted with water) is given to infants in our community from 6–7 months of age [20] but the practice may vary according to culture and the socio-economic status of the family. Further, cobalamin status of newborns and infants are positively associated with the breast milk concentration [5]. Specker *et al*. also reported that the cobalamin concentration in breast milk was positively correlated with maternal serum cobalamin concentrations which were lower in strict vegetarian women than in women on omnivorous diets [17]. In the current study, we didn't collect blood samples from the mothers; neither did we collect any dietary information or details on exclusive breastfeeding. However, in a previous study among women from the same community, we found that 83% had poor cobalamin status, as reflected in high MMA and low cobalamin concentration in plasma, and that 69% of the women consumed <1 µg of cobalamin per day [15] which is ∼50% of the RDA [4]. The poor cobalamin status observed in our study population is probably due to low cobalamin content in complementary food and/or poor cobalamin status of the mothers. Other contributing factors might be gastro-intestinal infections such as giardiasis which is common in developing countries and found in association with cobalamin deficiency [36].

The median plasma folate concentration in this study was 48.7 nmol/L which is approximately twice as high as in a study of a similar age group children in New Delhi [2] and it is interesting to note that none had plasma folate <5 nmol/L, the cut off which is frequently used to define folate deficiency [37] indicating that folate deficiency is not a problem in this population. In concordance with earlier findings [2], [32], [38], we also found higher plasma folate concentrations in breastfed and in children aged 6–11 months compared with older and non-breastfed children. In our study population, the low prevalence of folate deficiency may be due to the high proportion of breastfed children. The folate concentration is high in breast milk [39] which is, to a large extent, independent of folate status of the mother [40]. It may also be due to frequent consumption of foods like cereals, legumes and green leafy vegetables, which are rich sources of folate. We found that the mean plasma folate concentration was higher in the spring compared to in the summer season. This could be due to several factors, including that this season carries a relatively good availability of green leafy vegetables. In contrast to plasma cobalamin concentration, which reflects cobalamin intake over a long period of time, the plasma folate levels reflect recent dietary intake. Red blood cell folate status is recommended to reflect folate intake in individuals over extended periods of time [41]. However, we believe that this will not affect the overall mean folate status but can lead to unnecessary variability of the folate status indicator. It is also possible that the high folate concentration is due to the “folate trap” mechanism where the plasma concentration of folate is high because of poor cobalamin status [42].

### Strengths and Limitations

The main strength of the current study was its large sample size and that the data collection spanned over more than two years, including three hot and wet seasons. We also included measurements of the functional metabolic markers of cobalamin MMA and tHcy [43]. Our study included children with acute diarrhea, which is a selected child population. Incipient dehydration due to diarrhea at the time of blood collection may change the concentration of the biomarkers. In this study, 11% of the children had mild to moderately dehydration at the time of blood sampling, and we found that cobalamin and folate concentration were slightly higher in dehydrated children probably due to hemoconcentration. Also the use of non-fasting blood specimen may lead to under estimation of prevalence of folate deficiency. So the prevalence of cobalamin and folate deficiency among otherwise healthy children without dehydration might be even higher than our findings from children with acute diarrhea. Although, there were not any degradation of plasma MMA and tHcy, plasma folate concentration was degraded by 3% per year while storing at freeze [44]. Our plasma samples were stored for 3–4 years indicating that our estimate of plasma folate could be lower by 10–15% compared with the baseline values. We did not have all ages equally represented. Most of our study children were between 6–24 months old and breastfed (82%). The concentrations of these vitamins were associated with age, and our prevalence estimates might accordingly have been somewhat different if all ages were represented equally.

These limitations need to be kept in mind when drawing conclusions on the status of these vitamins in the general child population; however, we did not find any significant association of plasma cobalamin and folate with the duration and numbers of loose or watery stool. Nor did we observe any significant association with dysentery or vomiting episodes (data not shown). We did not obtain detailed information on dietary intake, exclusive breastfeeding pattern, the use of folic acid supplements by children and mother. Nor we were able to measure vitamin B6 which also can influence tHcy concentration [45]. Dietary intake may vary in different regions and in different ethnic groups. This information would have been useful for improving the interpretation of our findings.

### Generalizability

Our findings are to a certain extent generalizable to young children in other low-income countries with similar socio-economic conditions, and where the food habits are comparable. The present study was clinic based and included children with acute diarrhea. Most of the people in this study were from the Newar ethnic group and were non-vegetarian although regular intake of meat and fish was not common. Therefore we are uncertain whether or not our findings of prevalence of cobalamin deficiency can be generalized to all children and other parts of Nepal where the dietary habits may differ [46]. However, we believe that cobalamin deficiency is more prevalent in other communities of Nepal where vegetarian based foods are predominantly consumed. In a previous study, women belonging to the Chhetri ethnic group had significantly higher tHcy, MMA and lower cobalamin compared to the Newars [14].

### Conclusions

Cobalamin deficiency was common in children with acute diarrhea in this community, suggesting that this may represent a health problem. Breastfed infants had the highest prevalence of cobalamin deficiency. Future studies including healthy children need to be undertaken in order to expand on these findings and to explore consequences of poor and sub-optimal cobalamin status. If these finding are confirmed in other parts of Nepal, it may be justified to consider including cobalamin supplementation in a national strategy to improve nutritional status in children.

## References

[1] Bjorke-Monsen AL, Ueland PM (2011) Cobalamin status in children. J Inherit Metab Dis 34: 111–119. 20508991 10.1007/s10545-010-9119-1

[2] Taneja S, Bhandari N, Strand TA, Sommerfelt H, Refsum H, et al (2007) Cobalamin and folate status in infants and young children in a low-to-middle income community in India. Am J Clin Nutr 86: 1302–1309. 17991639 10.1093/ajcn/86.5.1302

[3] Allen LH (2008) Causes of vitamin B12 and folate deficiency. Food Nutr Bull 29: S20–34 discussion S35–27. 18709879 10.1177/15648265080292S105

[4] IOM (1998) Institute of Medicine. Food and Nutrition Board. Dietary Reference Intakes: Thiamin, Riboflavin, Niacin, Vitamin B6, Folate, Vitamin B12, Pantothenic Acid, Biotin, and Choline. Washington, DC: National Academy Press. 23193625

[5] Allen LH (1994) Nutritional supplementation for the pregnant woman. Clin Obstet Gynecol 37: 587–595. 7955646 10.1097/00003081-199409000-00011

[6] Trumbo P, Yates AA, Schlicker S, Poos M (2001) Dietary reference intakes: vitamin A, vitamin K, arsenic, boron, chromium, copper, iodine, iron, manganese, molybdenum, nickel, silicon, vanadium, and zinc. J Am Diet Assoc 101: 294–301. 11269606 10.1016/S0002-8223(01)00078-5

[7] Snow CF (1999) Laboratory diagnosis of vitamin B12 and folate deficiency: a guide for the primary care physician. Arch Intern Med 159: 1289–1298. 10386505 10.1001/archinte.159.12.1289

[8] Klee GG (2000) Cobalamin and Folate Evaluation: Measurement of Methylmalonic Acid and Homocysteine vs Vitamin B12 and Folate. Clinical Chemistry 46 8(B): 1277–1283. 10926922

[9] Vollset SE, Refsum H, Irgens LM, Emblem BM, Tverdal A, et al (2000) Plasma total homocysteine, pregnancy complications, and adverse pregnancy outcomes: the Hordaland Homocysteine study. Am J Clin Nutr 71: 962–968. 10731504 10.1093/ajcn/71.4.962

[10] Yajnik CS, Deshpande SS, Panchanadikar AV, Naik SS, Deshpande JA, et al (2005) Maternal total homocysteine concentration and neonatal size in India. Asia Pac J Clin Nutr 14: 179–181. 15927937

[11] Wald DS, Law M, Morris JK (2002) Homocysteine and cardiovascular disease: evidence on causality from a meta-analysis. BMJ 325: 1202. 12446535 10.1136/bmj.325.7374.1202 PMC135491

[12] Lindenbaum J, Savage DG, Stabler SP, Allen RH (1990) Diagnosis of cobalamin deficiency: II. Relative sensitivities of serum cobalamin, methylmalonic acid, and total homocysteine concentrations. Am J Hematol 34: 99–107. 2339684 10.1002/ajh.2830340205

[13] Chandra J (2010) Megaloblastic anemia: back in focus. Indian J Pediatr 77: 795–799. 20589460 10.1007/s12098-010-0121-2

[14] Bondevik GT, Schneede J, Refsum H, Lie RT, Ulstein M, et al (2001) Homocysteine and methylmalonic acid levels in pregnant Nepali women. Should cobalamin supplementation be considered? Eur J Clin Nutr 55: 856–864. 11593347 10.1038/sj.ejcn.1601236

[15] Chandyo RK (2002) Cobalamin and folate status among women of reproductive age in Bhaktapur, Nepal.

[16] Jiang T, Christian P, Khatry SK, Wu L, West KP Jr (2005) Micronutrient deficiencies in early pregnancy are common, concurrent, and vary by season among rural Nepali pregnant women. J Nutr 135: 1106–1112. 15867289 10.1093/jn/135.5.1106

[17] Specker BL, Black A, Allen L, Morrow F (1990) Vitamin B-12: low milk concentrations are related to low serum concentrations in vegetarian women and to methylmalonic aciduria in their infants. Am J Clin Nutr 52: 1073–1076. 2239784 10.1093/ajcn/52.6.1073

[18] Baker H, Frank O, Deangelis B, Feingold S, Kaminetzky HA (1981) Role of placenta in maternal-fetal vitamin transfer in humans. Am J Obstet Gynecol 141: 792–796. 7198383 10.1016/0002-9378(81)90706-7

[19] Allen LH (2005) Multiple micronutrients in pregnancy and lactation: an overview. Am J Clin Nutr 81: 1206S–1212S. 15883453 10.1093/ajcn/81.5.1206

[20] Ulak M, Chandyo RK, Mellander L, Shrestha PS, Strand TA (2012) Infant feeding practices in Bhaktapur, Nepal: A cross-sectional, health facility based survey. Int Breastfeed J 7: 1. 22230510 10.1186/1746-4358-7-1 PMC3285083

[21] CBS (2011) National Population and Housing Census 2011,Central Bureau of Statistics (CBS). Government fof Nepal. National Planning Commission Secretariat.

[22] Strand TA, Chandyo RK, Bahl R, Sharma PR, Adhikari RK, et al (2002) Effectiveness and efficacy of zinc for the treatment of acute diarrhea in young children. Pediatrics 109: 898–903. 11986453 10.1542/peds.109.5.898

[23] O'Broin S, Kelleher B (1992) Microbiological assay on microtitre plates of folate in serum and red cells. J Clin Pathol 45: 344–347. 1577973 10.1136/jcp.45.4.344 PMC495277

[24] Kelleher BP, Walshe KG, Scott JM, O'Broin SD (1987) Microbiological assay for vitamin B12 with use of a colistin-sulfate-resistant organism. Clin Chem 33: 52–54. 3542297

[25] Molloy AM, Scott JM (1997) Microbiological assay for serum, plasma, and red cell folate using cryopreserved, microtiter plate method. Methods Enzymol 281: 43–53. 9250965 10.1016/s0076-6879(97)81007-5

[26] Husek P (1995) Simultaneous profile analysis of plasma amino and organic acids by capillary gas chromatography. J Chromatogr B Biomed Appl 669: 352–357. 7581911 10.1016/0378-4347(95)00115-y

[27] Shipchandler MT, Moore EG (1995) Rapid, fully automated measurement of plasma homocyst(e)ine with the Abbott IMx analyzer. Clin Chem 41: 991–994. 7600701

[28] Bryce J, Boschi-Pinto C, Shibuya K, Black RE (2005) WHO estimates of the causes of death in children. Lancet 365: 1147–1152. 15794969 10.1016/S0140-6736(05)71877-8

[29] USAID/INACG (2002) Adjusting Hemoglobin Values in Program Surveys.

[30] De Onis M (2006) WHO child growth standards : length/height-for-age, weight-for-age, weight-for-length, weight -for-height and body mass index-for-age : methods and development. Geneva, World Health Organization.

[31] Hosmer DW, S L (2000) Applied Logistic Regression. New York: John Wiley & Sons Inc.

[32] Hay G, Johnston C, Whitelaw A, Trygg K, Refsum H (2008) Folate and cobalamin status in relation to breastfeeding and weaning in healthy infants. Am J Clin Nutr 88: 105–114. 18614730 10.1093/ajcn/88.1.105

[33] Karademir F, Suleymanoglu S, Ersen A, Aydinoz S, Gultepe M, et al (2007) Vitamin B12, folate, homocysteine and urinary methylmalonic acid levels in infants. J Int Med Res 35: 384–388. 17593867 10.1177/147323000703500313

[34] Davis RE, Icke GC, Hilton JM, Orr E (1986) Serum thiamin, pyridoxal, cobalamin and folate concentrations in young infants. Acta Paediatr Scand 75: 402–407. 3728001 10.1111/j.1651-2227.1986.tb10221.x

[35] Rimestad AH, Borgejordet Å, Vesterhus KN (2001) Den store matvaretabellen. (The Norwegian food composition table.) 2nd ed. Oslo, Norway: National Nutrition Council, 2001 (in Norwegian).

[36] Olivares JL, Fernandez R, Fleta J, Ruiz MY, Clavel A (2002) Vitamin B12 and folic acid in children with intestinal parasitic infection. J Am Coll Nutr 21: 109–113. 11999537 10.1080/07315724.2002.10719202

[37] Clarke R, Grimley Evans J, Schneede J, Nexo E, Bates C, et al (2004) Vitamin B12 and folate deficiency in later life. Age Ageing 33: 34–41. 14695861 10.1093/ageing/afg109

[38] Bjorke Monsen AL, Ueland PM (2003) Homocysteine and methylmalonic acid in diagnosis and risk assessment from infancy to adolescence. Am J Clin Nutr 78: 7–21. 12816766 10.1093/ajcn/78.1.7

[39] Mackey AD, Picciano MF (1999) Maternal folate status during extended lactation and the effect of supplemental folic acid. Am J Clin Nutr 69: 285–292. 9989694 10.1093/ajcn/69.2.285

[40] Murphy SP, Allen LH (2003) Nutritional importance of animal source foods. J Nutr 133: 3932S–3935S. 14672292 10.1093/jn/133.11.3932S

[41] WHO. (2012) Serum and red blood cell folate concentrations for assessing folate status in populations. Vitamin and Mineral Nutrition Information System. Geneva, World Health Organization.

[42] Scott JM, Weir DG (1981) The methyl folate trap. A physiological response in man to prevent methyl group deficiency in kwashiorkor (methionine deficiency) and an explanation for folic-acid induced exacerbation of subacute combined degeneration in pernicious anaemia. Lancet 2: 337–340. 6115113 10.1016/s0140-6736(81)90650-4

[43] Ueland PM, Refsum H, Stabler SP, Malinow MR, Andersson A, et al (1993) Total homocysteine in plasma or serum: methods and clinical applications. Clin Chem 39: 1764–1779. 8375046

[44] Hannisdal R, Gislefoss RE, Grimsrud TK, Hustad S, Morkrid L, et al (2010) Analytical recovery of folate and its degradation products in human serum stored at -25 degrees C for up to 29 years. J Nutr 140: 522–526. 20071651 10.3945/jn.109.116418

[45] Bostom AG, Gohh RY, Bausserman L, Hakas D, Jacques PF, et al (1999) Serum cystatin C as a determinant of fasting total homocysteine levels in renal transplant recipients with a normal serum creatinine. J Am Soc Nephrol 10: 164–166. 9890323 10.1681/ASN.V101164

[46] MOHP (2011) Ministry of Health and Population [Nepal], New ERA, and ICF International Inc. 2012. Nepal Demographic and Health Survey 2011. Kathmandu, Nepal: Ministry of Health and Population, New ERA, and ICF International, Calverton, Maryland.

